# Exploring Equity in Public Transportation Planning Using Smart Card Data

**DOI:** 10.3390/s21093039

**Published:** 2021-04-26

**Authors:** Kiarash Ghasemlou, Murat Ergun, Nima Dadashzadeh

**Affiliations:** 1 Graduate School of Science, Engineering and Technology, Istanbul Technical University, 34467 Istanbul, Turkey; 2 Civil Engineering Faculty, Istanbul Technical University, 34467 Istanbul, Turkey; ergunmur@itu.edu.tr; 3 Faculty of Civil and Geodetic Engineering, University of Ljubljana, 1000 Ljubljana, Slovenia; nima.dadashzadeh@fgg.uni-lj.si

**Keywords:** public transportation, smart card data, equity, cost benefit analysis, travel behavior, mobility pattern, transport planning, human centric planning

## Abstract

Existing public transport (PT) planning methods use a trip-based approach, rather than a user-based approach, leading to neglecting equity. In other words, the impacts of regular users—i.e., users with higher trip rates—are overrepresented during analysis and modelling because of higher trip rates. In contrast to the existing studies, this study aims to show the actual demand characteristic and users’ share are different in daily and monthly data. For this, 1-month of smart card data from the Kocaeli, Turkey, was evaluated by means of specific variables, such as boarding frequency, cardholder types, and the number of users, as well as a breakdown of the number of days traveled by each user set. Results show that the proportion of regular PT users to total users in 1 workday, is higher than the monthly proportion of regular PT users to total users. Accordingly, users who have 16–21 days boarding frequency are 16% of the total users, and yet they have been overrepresented by 39% in the 1-day analysis. Moreover, users who have 1–6 days boarding frequency, have a share of 66% in the 1-month dataset and are underrepresented with a share of 22% in the 1-day analysis. Results indicated that the daily travel data without information related to the day-to-day frequency of trips and PT use caused incorrect estimation of real PT demand. Moreover, user-based analyzing approach over a month prepares the more realistic basis for transportation planning, design, and prioritization of transport investments.

## 1. Introduction

The sustainable development of transportation has become a key point of interest on the part of scholars and policy-makers over recent decades. In this context, sustainability not only refers to environmentally friendly transportation systems but also to those that are economically and socially sustainable. In line with sustainable transport development, accessibility and mobility for all are two new paradigms altering conventional transport planning and policy-making. Sustainable mobility [1] and accessible transport [2] are not new concepts in transportation. For instance, bus priority methods are one of strategies to support sustainable and equitable transport planning by giving priority to bus on traffic congestion [3,4]. Beyazit [5] noted that sustainable transport and mobility plays a crucial role in distributing socio-economic benefits or losses, as well as social justice. Therefore, the efficiency of the exiting method regarding sustainable transport planning and projects analyses should be critically evaluated and improved considering issues such as social justice [5,6,7], equity [8,9], and social inclusion (exclusion) [8,9].

On the other hand, methods that are used in the evaluation and prioritization of public transport (PT) investment projects are more trip-based rather than user-based [10,11]. Using a trip-based approach, the needs of users with more boarding frequency are overrepresented in transport planning and modelling. In addition, some routes are used by more people overall, even though the number of users is rare on a daily basis. This being the case, the best example comes in the form of medical-based PT routes (trips to medical centers). The shortening or lifting of these PT routes, even if the number of trips is small, affects more people than the number of those using the line each day. This is because, along such routes, different users take the routes every day. On contrary, some routes have high boarding figures by day but are taken by the same users daily, such as commuter routes at rush hour.

The use of anonymous big data, e.g., PT Smart Card Fare Collection (PT-SCFC) data, has begun to figure more predominantly in the analysis of travel behavior and mobility patterns [12,13,14]. However, the misrepresentation and mis-modelling that characterizes the above in the form of using such large data can be found in a number of recently published studies [15,16,17,18,19]. In contrast to existing studies and considering the number of days and number of trips, or boardings that PT users make over a month, this study shows how more appropriate modelling can result in socially equitable opportunity and services to PT users.

In the following sections the statement of the art related to the equity in transportation and PT-SCFC application in PT is given. Afterward, a case study together with the associated data, and data-cleaning process is detailed. Then, results of the analysis and the related discussion on equity in PT planning is given. Finally, the highlights of the study together with the findings and suggestion for potential future studies is provided.

## 2. Background

### 2.1. Equity in PT Planning

Equity and social inclusion (exclusion) as a transport planning issue has been defined by Litman [20,21] as the fair distribution of benefits and cost impacts of all users. In addition, social exclusion (the transport of disadvantaged people) referred to the barriers (households do not own personal car, low-income individuals, persons with disabilities or reduced mobility, such as the elderly) preventing people from using public services like transport, education, and jobs, etc. It was concluded that inadequate transport planning, policies, and decisions have direct equity impacts and cause social exclusion. Transport equity and social inclusion analyses, in particular, in PT projects, are very difficult to conduct due to their complexity, spatiotemporal dimensions, impacts measurement, and users’ categorization [8,22,23,24,25,26]. There are also a number of psychosocial barriers to PT use when it comes to the elderly, who have specific need for a socially sustainable transportation system that should constitute a paramount consideration [27]. To address these challenges, three indicators proposed by Di Ciommo et al. [8], including: (i) indicators that make it possible to assess how much benefits or costs are being received by different population groups, (ii) indicators to disaggregate population groups from each other, and (iii) indicators to determine the equity of an observed distribution of a particular benefit or cost (e.g., transit subsidies or direct access to key activities). Equity in PT planning can be evaluated from different dimensions—namely, spatial and temporal dimensions considering socio-economic characteristics like age, gender, income, and health condition (e.g., disability).

- Socio-spatial equity: equitable access to transport services in terms of spatial and land-use patterns [28,29], such as transport accessibility in urban area vs. sub-urbans or rural area, as well as in CBD (central business district) area vs. non-CBD area;
- Temporal equity: equitable access to transport services considering the time-critical nature of accessibility needs [24], such as transport accessibility for users with low trip frequency or high trip frequency (regular users) during peak-hour or off-peak-hour.

To evaluate PT transport projects, Cost Benefit Analysis (CBA) is one of the most common techniques [10,11,30,31,32]. There are also other project appraisal methods such as the Multi Criteria Analysis (MCA), social-based analysis, decision-analysis, simulation, and mathematical modelling. CBA has some limitations, such as the complexity of the concept in application and the weakness in assessing environmental, health, and social issues, such as equity and social justice—as these elements cannot be examined in monetary terms [5]. For instance, it has been found that the CBA framework and project appraisal considering only “travel time savings” has various equity effects [10]. The results also show that CBA appraisal are not accurate enough to measure distributional impacts of equity.

### 2.2. Smart Card Data Application in PT Planning

PT smart card data benefits scholars and practitioners in understanding urban dynamics and human activities [12,33]. For instance, smart card data can be used to estimate the Origin-Destination (OD) of PT users, long-term network planning, demand forecasting, operational purposes like timetable and schedule adjustments as well as PT funding and investment decisions [13,33,34,35,36,37,38,39,40]. PT smart card data includes information on boarding numbers, their times, and locations. As tons of data are gathered by fare collection systems every day, data exploration and data-driven segmentation on users has been become an essential issue. Data-driven segmentation analyses consist of spatial and temporal probability. The first refers to users’ tendency to board a given station for their trips, while the later refers to users’ tendency to travel at a particular hour of the day i.e., trip frequency [41]. Espinoza et al. [42] measured PT users’ behavior change in travel over time by splitting data into different time windows using three algorithms (Transition Probability Matrix (TPM), Spatiotemporal Edit Distance Method (EDM) and Regions of Interest and Feature Vector (RoIs-FV)). It was found that the results obtained for the same users can be different using different algorithms. However, the behavior of the variability through time is similar for the three algorithms evaluated.

Most existing studies on the smart card data evaluation are provided on a daily bases and there are very limited studies on the monthly trip frequency of PT users. Benenson and Ben-Elia [43] have illustrated the unexpected flexibility of PT usage by considering their daily boarding numbers. This study has examined data on a weekly basis only and focuses on trip counts alone. The results find that the rate of trips made once or twice in the total weekly data are higher than expected. Some studies considered the variability of demand on PT and temporal rhythms in travel and activity patterns [42,44,45,46,47]. However, the actual PT demand is difficult to be obtained as it changes continuously over the time (period of the day, day of the week, season, or holiday) [48]. For instance, Morency et al. [47] showed that spatial and temporal variability of PT use can be measured using smart card data considering bus stops used for boarding and frequency of using of the bus stop. K-mean algorithm was used to cluster transit use cycles and homogenous days and weeks of travel among card segments and at various times of the year. Likewise, Raux et al. [45] have used the sequential alignment method in measuring the variability in day-to-day travel-activity behavior according to interpersonal and intrapersonal differences of attributes. They concluded that intrapersonal variability is greater than the interpersonal one considering the random part of behavior, while intrapersonal variability is lower than the interpersonal one due to habitual part of behavior.

Liu et al. [49] underlined the relationship between user demographic characteristics (e.g., the role of gender and age) and the variability of travel behavior. Results showed that female users exhibit higher intrapersonal variability than their male counterparts. Weekly patterns are the most diverse for users aged 70+, followed by the users aged 65–69. More recently, Egu and Bonnel [44] combined clustering algorithm with day-to-day intrapersonal similarity metric to explore day-to-day intrapersonal variability of PT usage. It was found that there is no one-size-fits-all approach to the problem of day-to-day variability of transit usage. According to their studies, low-frequency users represent only 1% of the total journey, although it is 14% of the total data. This study is not related to determination of real demand of PT users and equity issues. Clustering method used in the existing studies caused losing some of information related to day-to-day individual behavior, and they focused more on most common day-to-day usage pattern results.

The literature review shows that in studies based on smart card data, there was no comparison in terms of overrepresented and underrepresented users and trips between whole data and 1 day data. Therefore, this study aims to show the importance of defining temporal frequency to better determine the actual share of users and demand in transport planning. We define a new approach in equity analysis of transport planning that reveals the actual demand characteristic, and users’ share are different in daily data (which has been usually used by the existing transport planning methods) compared to the real demand and users’ share. Thus, our study has purposefully focused on the trip and PT usage frequency of users to be extracted from a 1-month set of PT-SCFC data. These are the main contribution of our study with respect to the related work.

## 3. Material and Method

### 3.1. Data and Study Area

Kocaeli is one of the most populated provinces in Turkey, with a population of around 1.9 million (2019) across 12 districts, 13 municipalities, including one metropolitan and 12 district municipalities, and a population density of 575 people per km^2^. Briefly, 93.7% of the population live in cities, while the rest live in villages. Kocaeli Metropolitan Municipality (KMM) is located at the easternmost end of the Marmara Sea around the Gulf of Izmit [50].

Public transport is one the most frequent transportation modes, with a daily average ridership of around 408,000 passengers. Among motorized transportation, PT share represents 37% of the total modal share [51]. Information about the data on the mode share of PT according to trip purpose is given in Table 1.

**Figure 1:**
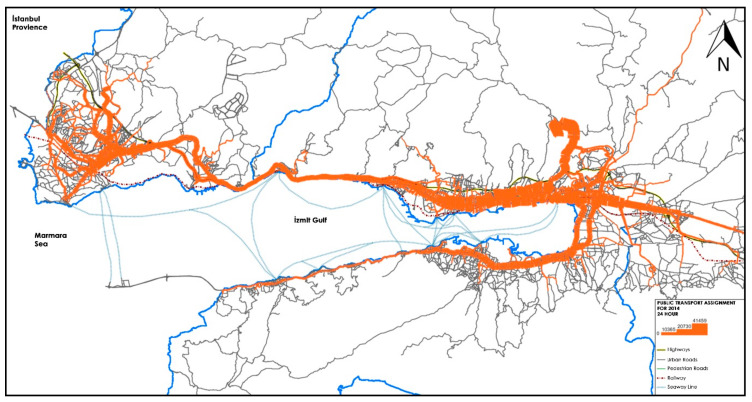
Public transport (PT) network assignment in our study area; Kocaeli metropolitan municipality.

There are 361 official bus routes in KMM, but 293 of these currently are active and operational. KMM’s bus transport network includes a total of 7400 bus stops, but the maximum number of active bus stops comes to 4869. Figure 1 shows the study area and PT assignment results, which is modelled using VISUM macroscopic transport modelling software [52].

In this study, the smart card data from the KMM were used. The dataset included 1-month smart card data of November 2018. The PT smart card data of KMM included unique card ID, card types such as normal, student, elderly, and people with disabilities cards, boarding station ID, card boarding (validation) time, cost boarding, type of boarding, route, direction, and vehicle ID. When the user boards the buses, the smart card’s fare was validated. The card validation process consisted of the following steps: the Global Positioning System (GPS) reader on the bus identifies the stop where the boarding is made. The system validates the run (correct route) at this location as the bus system contains planned runs for a single day (a run refers to a sequence of stops to be deserved; it usually represents one direction of a route). Card numbers, dates, times, validation status, and stop numbers are stored at each boarding. This information is downloaded to the central server at of the end of each day. Smart card boarding data was collected over a period of 30 days for each card. Each record was divided into nine binary variables. This day division was common to transport planning studies in Turkey, though other countries may have different time intervals.

The data set was a compilation of 12,250,983 boarding validations made by 808,834 card holders on the Kocaeli Public Transport network between 1 November and 30 November 2018. The average daily number of users came to 201,212 people with a standard deviation of 35,155. Sunday November 14th had the minimum number of users with 121,711 (240,461 boarding), while Thursday November 18th had the maximum number of users by 227,347 (466,280 boarding). As the boarding numbers vary on weekdays and weekends, it would be more accurate to analyze them separately.

### 3.2. Data Analysis Method

**Figure 2:**
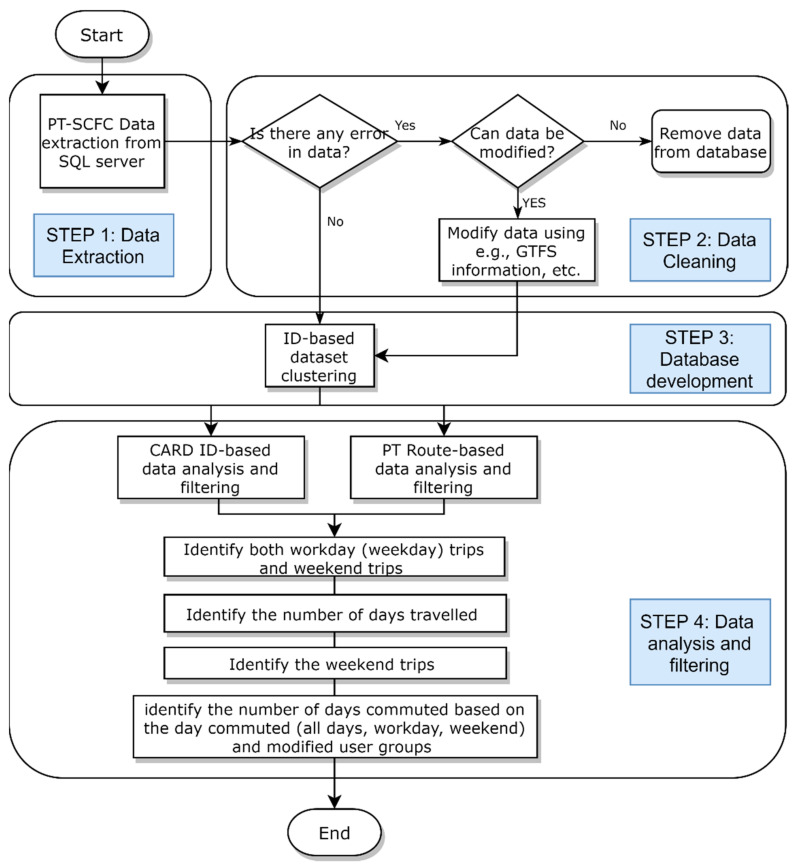
Flow chart of the data mining and categorization in this study.

A monthly smart card dataset with information on around 12 million raw boardings (validation) extracted from a SQL Server in KMM was obtained. Then, Python and Excel were used for tracking each unique ID of card holders in the ensemble of the data set. The analysis was conducted as an aggregate view of 808,834 card holders. Then, various data mining techniques and tools (see [53], for more information) were used to clear and prepare the dataset align with the objectives of this study. The steps of data analysis in our study were as follows: data extraction from SQL server, data cleaning, dataset development, data filtering, and the categorization of both Card ID-based and PT route-based categorizations, as seen in Figure 2. Looking at the flowchart, data cleaning and categorization algorithm procedures succeed in the following:

**Step 1. Data Extraction**

1.1. Extracting data from SQL server of KMM;
1.2. Converting extracted data to daily basis Excel file;

**Step 2. Data Cleaning**

2.1. Identifying the data without coordination information;
2.2. Identifying the data with false coordination information;
2.3. Modifying data coordination information according to General Transit Feed Specification (GTFS) data coordination (PT schedules and associated geographic information provided by Google);
2.4. Categorizing card holders into six user groups with similar characteristics to come across more meaningful analysis.

**Step 3. Data set development and clustering**

3.1. Filtering the dataset based on ID numbers;
3.2. Extracting the following information for each ID (card holder) and add to ID dataset: daily number of boarding, trip-day information (which day and total number of days in 1 month), and card type. This refers to the calculation of group characteristics of each cardholder ID per day (frequency of PT use, boarding rate per workdays and weekends) and the calculation of its average values per user (including the average frequency of use and boardings per workday etc.);
3.3. Clustering natural cardholder groups according to their average values;
3.4. Clustering cardholder frequency groups (30 groups according to number of day that cardholder used PT);
3.5. Creating PT route-based dataset and extracting the boarding data of bus routes.

**Step 4. Data analysis and filtering**

*4.1*. *CARD ID-based data analysis and filtering*
  4.1.1. Identifying both workday (weekday) trips and weekend trips in ID-based dataset, and assign a dummy variable value (0 and 1) to them;
  4.1.2. Identifying the number of days commuted by every card holder ID in entire dataset, determine the average daily trips, average monthly trips, and their standard deviation (STD);
  4.1.3. Identifying the workday trips by every card holder ID, in entire dataset, determine the average daily trips, average monthly trips, and their STD;
  4.1.4. Identifying the weekend trips by every card holder ID, in entire dataset, determine the average daily trips, average monthly trips, and their STD;
  4.1.5. Identifying the number of days commuted by PT based on the day commuted (all days, workday, and weekend) and modified user groups (6 clustered user groups), determining the average daily trips, average monthly trips, and their STD.
*4.2*. *PT Route-based data analysis and filtering*
  4.2.1. Filter the data based on PT routes;
  4.2.2. For each PT route, identify the workday trips and weekend trips in entire ID dataset, and assign a dummy variable value (0 and 1) to them;
  4.2.3. For each PT route, identify the number of days commuted by every card holder ID in entire dataset, determine the average daily trips, average monthly trips, and their STD;
  4.2.4. For each PT route, identify the workday trips by every card holder ID in entire data set, determine the average daily trips, average monthly trips, and their STD;
  4.2.5. For each PT route, identify the weekend trips by every card holder ID in entire dataset, determine the average daily trips, average monthly trips, and their STD;
  4.2.6. For each PT route, identify the number of days commuted based on the day commuted (all days, workday, and weekend) and modified user groups (i.e., six similar card holder groups), determine the average daily trips, average monthly trips, and their STD.

To apply group characterization, first the whole data set (1 month) is considered as an ensemble data set. Then, the raw datasets were subdivided into large, homogeneous clusters on the basis of trip patterns observed over a monthly basis. The goal was to split users in homogeneous groups according to their behavior and illustrate the similarity between each group. Afterward, card holders were categorized according to the number of days they used PT. For this, the following equations were defined to examine the impact of user groups’ frequency and to detect this group of users in data set.

(1) Percentage of card holder groups in 1-month dataset=SCg,i,WD∑i=1nSCg,i,WD

(2) Percentage of card holder groups in 1-day dataset=i*SCg,i,WDn∑i=1ni*SCg,i,WDn

(3) Percentage of card holder boardings in 1-day dataset=i*TCg,i,WDn∑i=1ni*TCg,i,WDn,

where

- *WD*: notation for weekday or weekend/holiday; whether the day of boarding (boarding) is weekend or holiday; *WD* = 0, else *WD* = 1;
- *SC_g,i,WD_*: number of card holder of group “*g*”, which have boarding in “*i*” days in weekday or weekend/holiday;
- *TC_g,i,WD_*: number of boarding of card holder of group “*g*”, which have boarding in “*i*” days in weekday or weekend/holiday;
- *n*: number of days; for 1 month, *n* = 30; for weekday, *n* = 21; and for weekend, *n* = 9.

Later, we examined the variability of the group belongings to 1 month of observation. This provided us an initial idea about the regularity of the habits over time. It also identified unusual weeks in terms of travel behavior.

In this stage, the objective was to determine more “natural” groupings of users in order to split them into homogeneous groups according to behaviors and to show the comportment of each group. In the raw dataset extracted from the SQL database, there can be seen various types of PT smart cards, namely, 01: Normal, 10 Normal with Credit Card, 04: Student, 05: Reduced fare card, 06: Teachers, 07: Bus drivers, 16: new card for aged 65+, 65: aged 65+, 75: Disabled with Accompanying, 74: Disabled, 13: Limited-use Card (1–5 boarding limit), 66: Local Press Card, 67: National Press Card, 68: Staff Card, 69: Free of charge type 1, 70: Postman Card, 72: Allowance card for employees, 73: Trainee Card, 76: Temporary Staff, 77: TUIK (Turkish Statistics Office) employee, 78: Veteran and Martyr Card, 79: Municipality parking staff, and 87: Free of charge type 2.

According to the sample dataset, PT users were reclassified and clustered through a data mining method according to their card types’ characteristics, namely, 1: Normal, 2: Student, 3: Elderly, 4: People with Disabilities (PwD) and their accompany people/relatives, 5: limited-used, and 6: others. The number of groups is fixed according to the usage frequency of PT during workdays within the month.

Lastly, we carried out a route-based analysis on users in terms of card type, number of boarding per day, number of unique card holder per day, and monthly frequency of usage (in workdays) over the course of 1 month.

## 4. Results and Discussions

### 4.1. Group Categorization Analysis

Table 2 presents the original card types and revised card types, and their boarding (boarding) information.

It was expected that the card holders of type 6 (Teachers) and type 4 (Students) would be categorized in the same group, but according to their average frequency of PT usage over the course of 1 month (workdays and weekends), it was found that the travel behavior was different. For example, students had an average monthly 22.3 boarding per person, while teachers had 18.1. In addition, the frequency of days in which students used PT came to 12.2 per month, while this value for teachers came to 10.4 per month.

**Figure 3:**
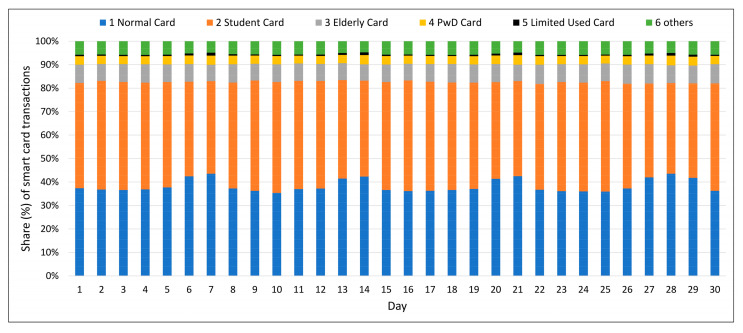
Distribution of boarding by different user groups.

In this regard, Figure 3 illustrates the share of user groups based on their smart card boarding per day, while Table 3 presents the share of user groups based on their smart card boarding on weekly basis.

It is evident that the total number of smart card boardings according to a particular user group differs in each day of the week based on user group. Table 3 also summarize the distribution of card holders in weekdays and weekends.

In one hand, it is clear that holders of card type 1 (Normal or Adult card) and type 5 (1–5 boardings) take a higher share of the weekend boarding data (type 1: 42.3%, type 5: 1.0%) compared to their share in weekdays boarding data (type 1: 36.6%, type 5: 0.5%). On the other hand, it can be seen that student card holders have a larger share during the week.

In terms of average number of boardings, card type 3 (Elderly card holders) and card type 4 (PwD card holders) have significantly higher boarding rates on weekdays and weekends. This shows the possibility that these users demand more travel. It is also possible that the transportation system is not planned well enough for this group of the users and they have to travel considering more transfer between bus routes.

In terms of the use of PT boardings data to estimate the OD, the question of whether this boarding rate is due to the journey or the transfer between bus lines can be gleaned. However, this is beyond the scope of our study and further studies to this end are suggested in Section 5.2.

### 4.2. Monthly’s Trip-Frequency Analysis on User Groups

The data of smart card users according to three parameters was analyzed—namely, trip frequency over the month, the boarding rate of users per day, and the type of card. For this, users are categorized according to the number of days, which they boarded over the course of 1 month.

**Figure 4:**
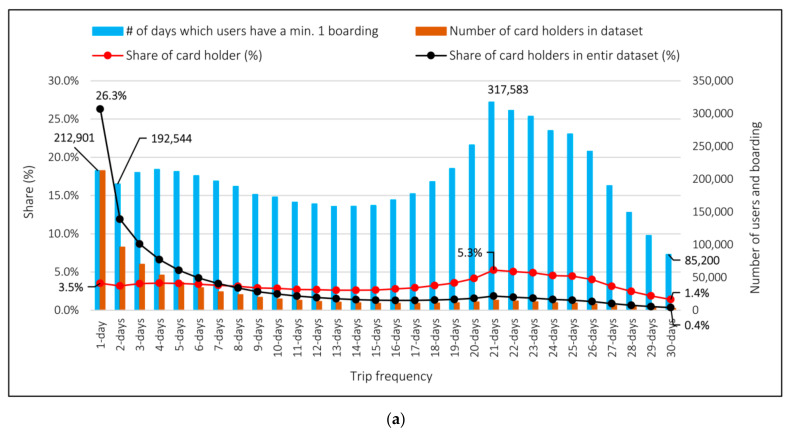

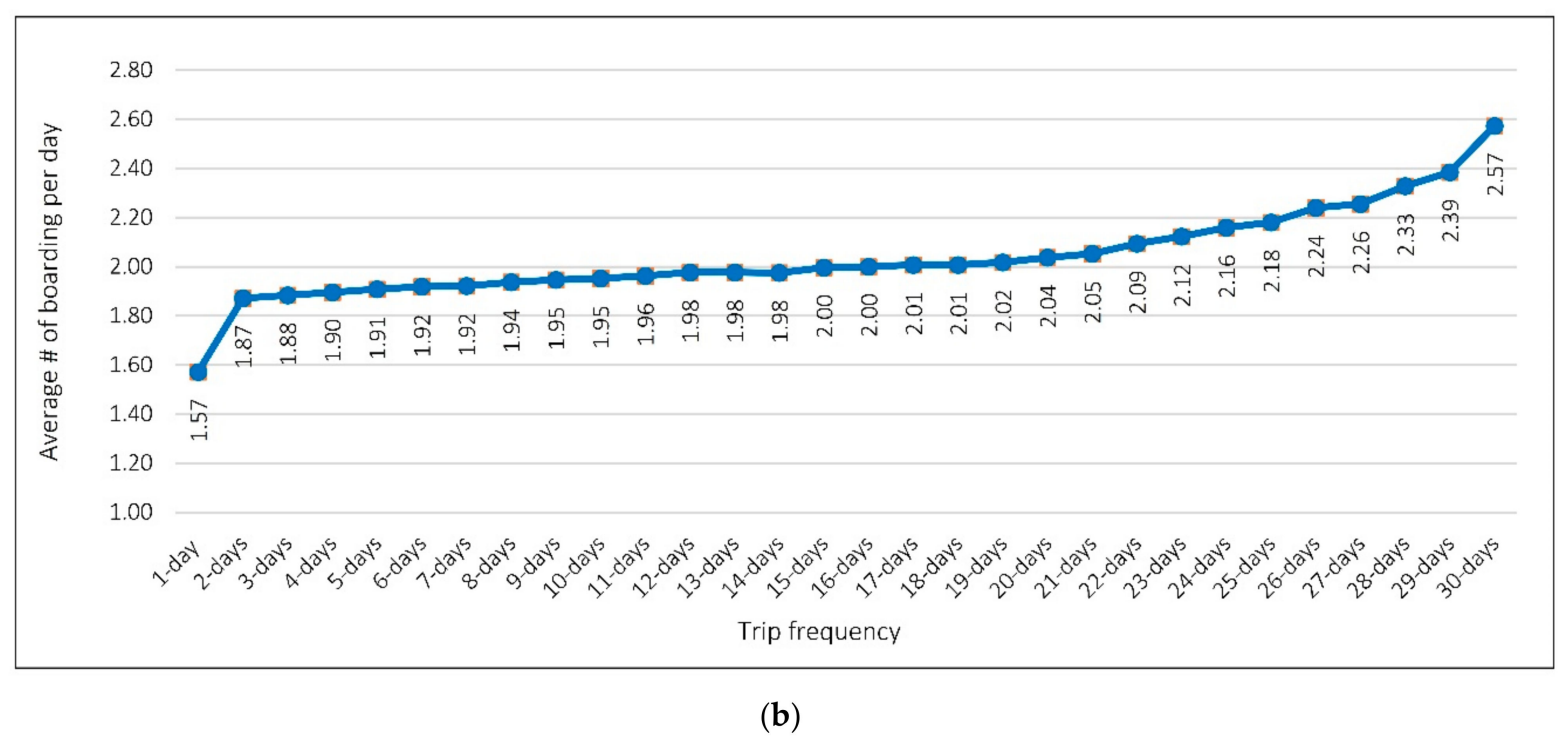
(**a**) Number and share of card holders and boarding and (**b**) average boarding per card holder of each group.

**Figure A1:**
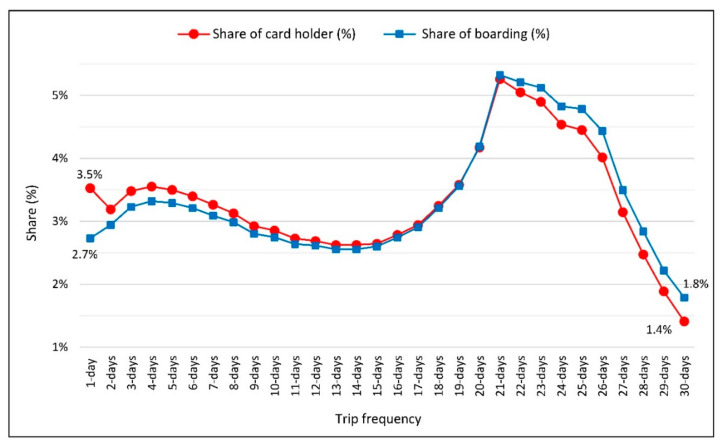
Share of card holders vs. share of boarding over a month.

Figure 4a illustrates the distribution of user groups and their boardings over the month, according to their trip frequency, in which the 1-day trip-frequency group denotes those who traveled on only 1 day in the month, while the 30-days trip-frequency group represents those who traveled every day in a month. Figure 4b depicts average number of boarding per day by each user in different trip-frequency groups (See Appendix A; Table A1 and Figure A1 for more information).

As can be seen from the distributions in Figure 4a, users with less frequency are seen less in the total data, users with 1–6 days frequency and 63% have 21% ratio in the 1-day average data. On the other hand, Figure 4b shows that users that have minimum 1 boarding over workdays have 1.57 boarding per day on average. The maximum boarding number comes to 2.57 on average per day, which is related to users who have taken the PT vehicles every day of a month. This is due to the fact that users with low trip frequency have a lower boarding rate in only 1 day of data. In terms of trip frequency, they will be seen much less frequently as they have lower rates. In addition, it can be concluded that it would be more accurate to divide the total data in terms of weekdays and weekends.

### 4.3. Workdays’ Trip-Frequency Analysis on User Groups

Weekend travel behavior varies more than that of weekdays due to increased variations of the purpose of trips (i.e., since there are no work or school-based trips at the weekend), as well as a reduced number of active buses and the number of active bus lines. In our case, the rate of users who did not travel on weekends came to 36% (64% traveled the weekend). The share of weekend trips came to 22.48% of the total. The share of weekday trips, meanwhile, corresponded to 88.72%. Users who traveled both on weekdays and weekends (64%) composed of 9.37% of total users and 8.14% of total boarding data. Users who traveled only on the weekend came to 11.3% of total users, which corresponds to 8.1% of total weekend boardings and 1.8% of total boarding data.

Thus, user behavior analysis should be conducted for workdays and weekends separately. Yet this study focuses on workday trips in particular. Table 4 presents the share of boarding and number of users for each trip-frequency group.

Based on the results of Table 4, it is evident that user groups with low frequency have lower boarding rates compared to other groups, and this rate increases as frequency increases. In addition, users who travel 16–21 days on average during workdays corresponds to 42.6% of users who traveled every day and 49.14% of boarding data. It is also seen that users with low fecundity (traveling for 1–6 days) represent 22.14% of average daily users and 22.04% of boarding data.

Considering user distribution over the month, it is clear that the majority of active users are in 1–6 days trip-frequency groups (those who travel 1–6 days per month). On the other hand, it is clear that high trip-frequency users carry a higher share in the total data. The reason for this is that these group of users travel more days of the week and have a higher number of daily rides. The distribution of user groups and their boardings per day is given in the graph below, obtained from the workdays trip-frequency analysis (see detailed results in Appendix A; Table A2 and Table A3).

The proportion of regular PT users out of the total in 1 workday is higher than that of total users in our 1-month dataset. For instance, the card holders which have 1–6 days boarding frequency have a share of 66% in the whole dataset, while they would be underrepresented with a share of 22% according to a 1-day analysis. Moreover, card holders with a 16–21 days boarding frequency came to 16% of all cardholders and would be thus overrepresented as 39% in 1-day analysis.

**Figure 5:**
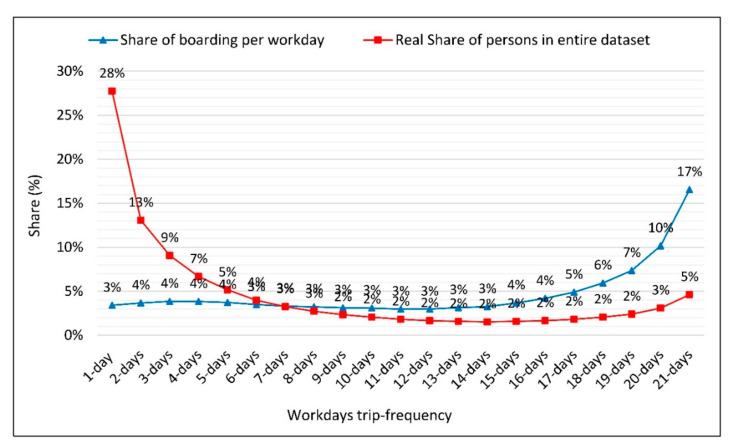
Share of boarding per workdays (blue) vs. real share of persons (red) in entire dataset.

As shown in Figure 5, the share of users with low frequency has low boarding rate, while users with high frequency have a high rate of the total workdays boarding. Moreover, it is clear that users’ frequency increases with a rise in the average number of boardings. When cardholders are evaluated on the basis of defined groups, the relevant weekday trips and user shares are given in the graphs below. In this study, the coefficient of variation (CV) as a statistical measure was used to evaluate the variation of data distribution around the mean of data and calculated by dividing the STD by the mean. Depending on the field of research, the acceptance values of CV vary between <5% (medicine) and <20% (engineering) [54,55]. When CV values are assessed in our case, the values came to over 20% only for card holders of type 2 (students) with a trip-frequency of 1- and 2-day out of all workdays over the month. These trip-frequency groups represent a small share (0.85%) among all boarding data. Therefore, it is assumed that the mean represents this group. Please see Appendix A Table A3 for more detailed information.

**Figure 6:**
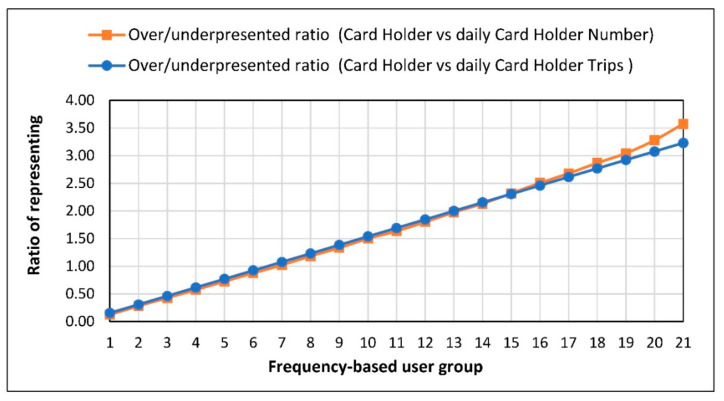
Raito of workday share to 1 month share of each frequency group.

To highlight how frequency-based user group boardings and cardholder share can be over/under representing (over-weighting and under-weighting) more clearly, the division of the real share of each group in single-month data for workdays is shown in Figure 6.

As seen in Figure 6, groups of users with a higher frequency of use are more overrepresented, while groups with a lower frequency of use are more underrepresented. In addition, it can also be observed that higher user groups have a higher number of boardings per day (causing a higher more share than in 1-day data) and are significantly more overrepresented.

User groups with lower frequency are under-represented because they are less likely to appear in a day’s data, and on the contrary, user groups with high frequency are more likely to appear in a daily data. For example, the probability of a user group traveling only one day to be seen in one day is 0.48 (1 day/21 workdays). However, the user group traveling every day (the group traveling for 21 days) will definitely be seen on any selected working day and the probability will be 1. Consequently, user groups with a high probability of being seen when looking at the selected daily data are represented much more than their shares in the total monthly data.

The representation shares of different frequency groups in 1-day data are directly related to their shares in 1-month data. In this context, although the shares of the data used in this study and the public transport data belonging to the other center are different depending on the user behavior, the representation tendencies in the 1-day data will be similar to the trends in this study.

By considering Figure 4b, higher frequency groups have higher probability of seeing in a daily trip data when a comparison is made between daily travel and real user shares of these groups, because of having higher trip rates in each day’s addition to higher probability related to their frequency of using PT. On the contrary, due to the fact that low-frequency groups also have lower number of trips, their share in 1-day data are much less represented than their user share in 1-day data.

**Figure 7:**
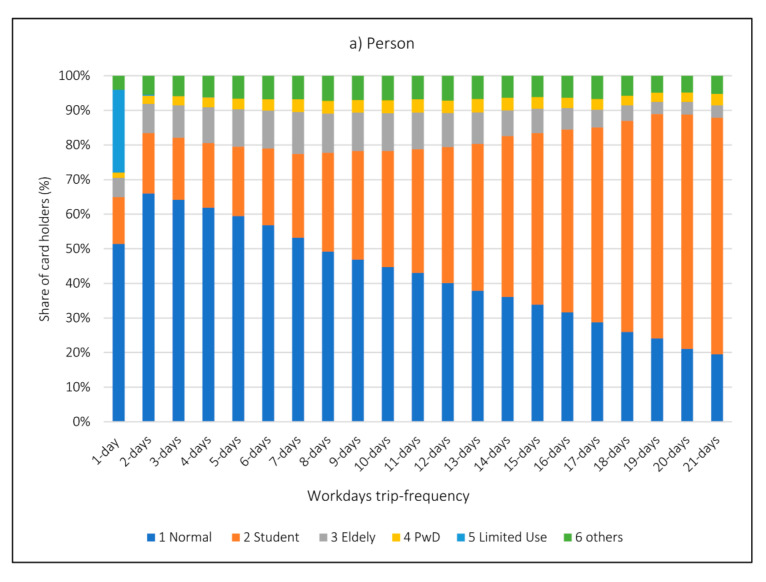

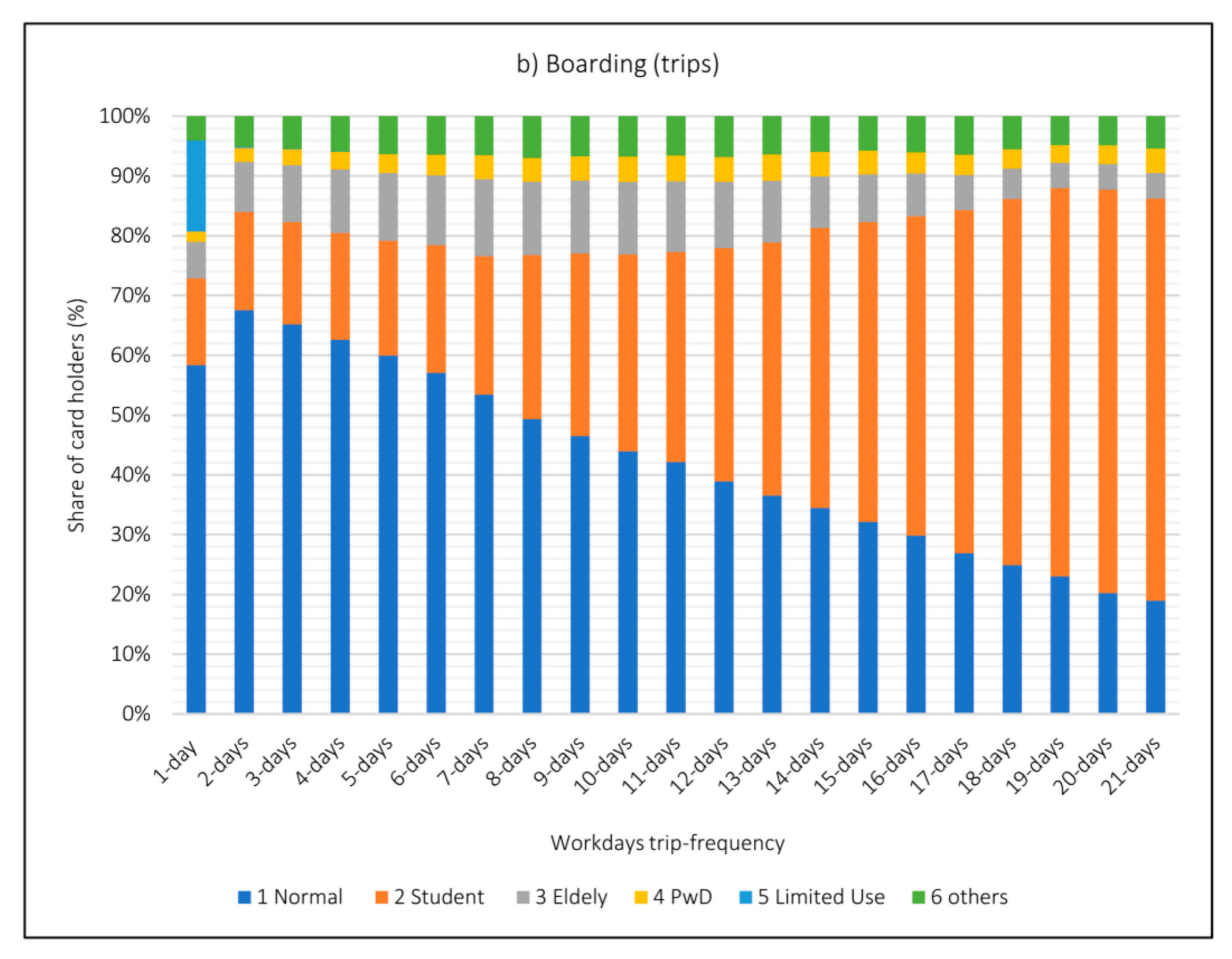
Share of different user groups on workdays in a month according to: (**a**) number of persons and (**b**) number of boardings in each user group.

As shown in Figure 7, different card holder groups have varying trip frequency behavior. Card holders from type 1 have the highest share among low frequency types, while card holders of type 2, behave exactly in the opposite way and higher frequency group of users have a higher share in terms of boardings and users. Card holders from type 3, the group of 7-day frequency, showed the highest share among other frequencies of this type of card holder. Card holders type 5 (1–5 limited boarding), are mostly user groups with a frequency of 1 day on workdays. Card holders type 4 and 6 have significantly similar distribution rate among trip frequency groups.

User groups which are in higher frequency groups are users who travel on regularly bases. This means that as share of high frequency group of card holders increases, the probability of being seen in a day’s data will also increase. In this context, the share of student card holders is increasing steadily with more frequent user groups. They have 68% share of higher frequency groups, which are traveling every day. It means that more regular use of public transportation are students and educational purposed trips that more regularly occur by public transport when compared to the purposed trips. It is also possible to say that the students are represented more in one day PT data than their actual shares in PT users. Yet, the opposite is true for Normal card users, and the share of student card holders is decreasing steadily in more frequent user groups. Accordingly, the share of these user groups decreases up to 20% among the users who travel every day. It is concluded that the regular users’ purposed trips (other than educational trips) are underrepresented in a day’s public transport data.

It is seen that different card users and their travel shares change within the Frequency groups. It means that especially users with higher frequency are users who travel regularly. The probability of a cardholder type to be seen in a day’s data increases as the tendency to use regular public transport increases.

In general, and as one of the main hypotheses suggested by this study, it is shown that an evaluation of users’ daily behavior is not enough and that different user groups (with different trip-frequency) show different travel (boarding) patterns.

### 4.4. Bus Line-Based Analysis on Workday Trips

Moreover, we have also carried out a bus line-based analysis using 1-month data to understand and show more concrete evidence regarding the (in)efficiency and impact on PT services planned. Hence, in each PT line such as bus, rail, and ferry lines, the types of card holders and their monthly boarding frequency (how many boarding/day) have been examined.

All of the 293 KMM’s PT lines, two bus lines—namely, lines no. 118 and no. 23, with share of 1.45% (rank 14) and 1.41% (rank 15) of total PT ridership, respectively—have been selected for further analysis and comparison. Both of which are serving users in KMM districts that also have Tramway lines (a light rail PT system). Users and boarding information of the selected bus lines is as follows:

- Bus lines no. 118 and 23 have on average 6348 and 6802 boarding per workday, respectively.
- Average daily card holder numbers of these lines are 5014 and 5531 per workday, respectively.
- The total number of card holders per workdays are 38,628 and 45,921 for bus lines no. 118 and no. 23, respectively.

The main reason to select these bus lines is to show that although both have similar numbers of users and boardings on a daily basis, they have totally different characteristics. For instance, bus line no. 23 is more frequently used by students, while bus line no. 118 is more frequently used by normal cardholders. Furthermore, line no. 118 is more frequently used by the Elderly and PwDs card holders than line 23 and the frequency of use by card holders varies between them. As a result, the number of daily passengers (cardholders) and daily boardings will not be enough to determine the real number of users of each line. Line 23 has more daily boardings and users (cardholders); however, line 118 is used more frequently over the course of the month.

**Figure 8:**
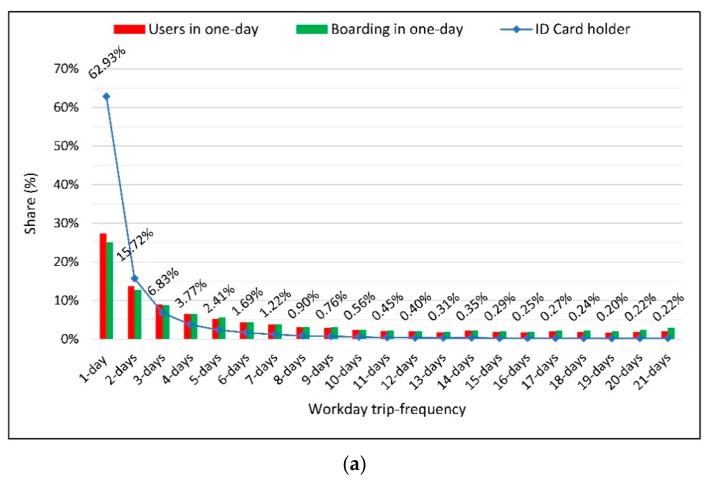

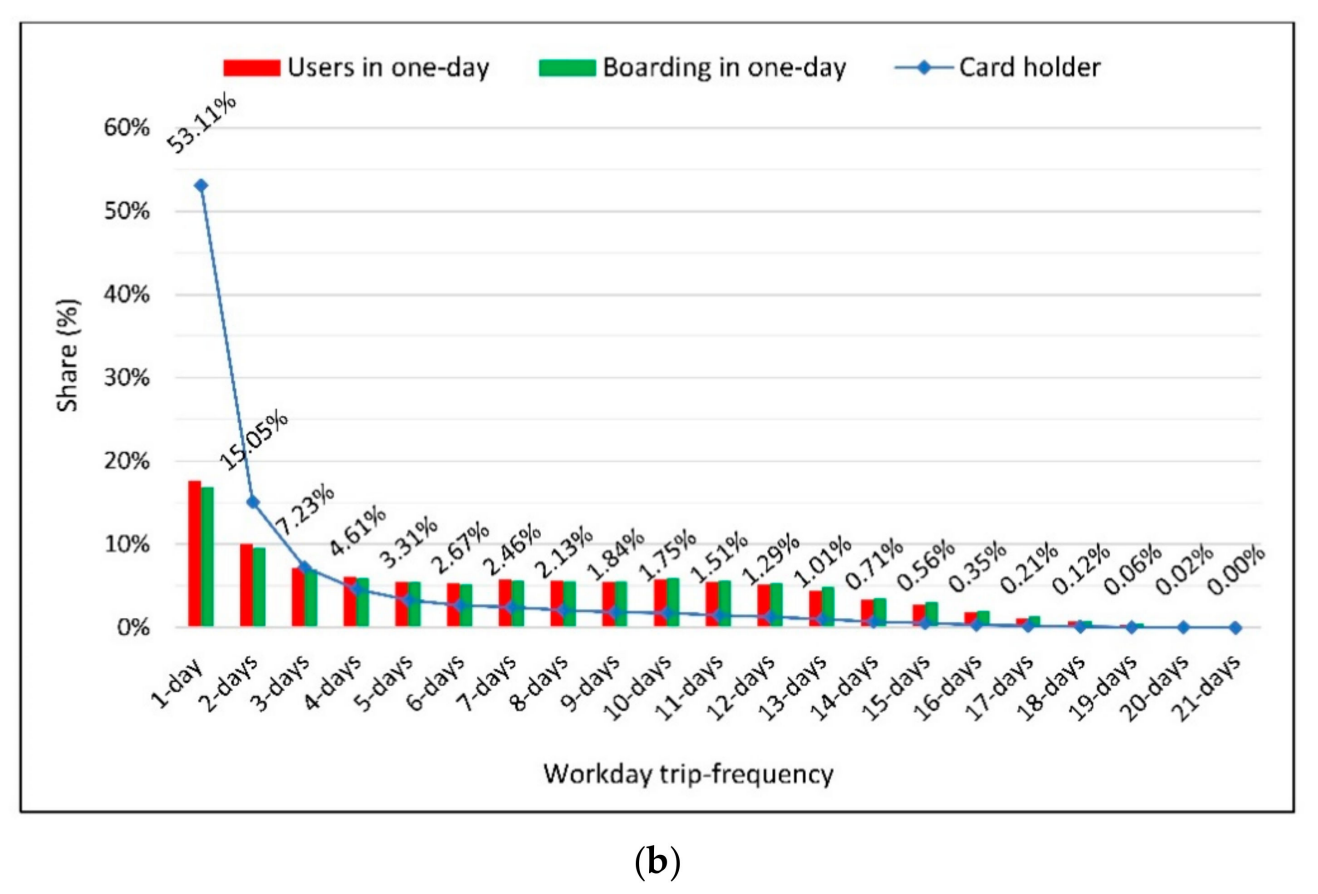
Comparing 1-day data and 1-month data in terms of number of users and boarding for: (**a**) Bus Line No. 118 and (**b**) Bus Line No. 23.

In other words, the number of boardings and users on bus line no. 118 comes to less than the number of boardings and users on bus line no. 23. Meanwhile, considering the monthly number of users, bus line no. 23 serves more PT users (45,921) compared to bus line no. 118 (38,628). The distribution of users and their boarding frequency over workdays over a month are given at Figure 8a (bus line no. 118) and Figure 8b (bus line no. 23).

In Figure 8a,b, it is seen that the user groups with lower frequency in Line 118 are using this line more than line 23. In this context, the share of passengers using line 118 less than 4 days in a month are 85%, while the share of these users using line 23 is 75%. Consequently, although the number of real users in this line are more than line 23 in the monthly data, line 23’s number of users and travel in daily data are higher.

Table 5 presents the distribution of daily users and daily boarding among different card type holders, and the real number of users are using bus lines no. 23 and no. 118 in a month.

As given in Table 5, card holder type no. 1 is underrepresented in daily data for both bus lines. For instance, in bus line no. 23, normal card holders have a share of 23.3% of all daily users, while the real share of card holders type no. 1 represents 34% of monthly users.

On the other hand, all other card types, in particular students (type no. 2), are overrepresented in daily data. In bus line no. 23, student card holders have a share of 70.7% among daily users, while the real share of these users among monthly users comes to 56.8%.

This situation approves the hypothesis of the study in terms of line-based evaluation as well. Therefore, taking only the daily number of users and boarding as a performance measure/criterion causes misleading evaluations of real performance due to the variation (distribution) of users and boarding frequency over a month.

## 5. Conclusions

Generally, PT systems’ evaluation and planning are more trip-based rather than user-based, e.g., in conventional methods such as cost benefit analysis, multi criteria analysis, and social-based analysis, which causes a miscalculation (overestimation). In other words, the needs of users with more frequency outweigh and are overrepresented in conventional transport projects analyses. To address this mis-estimation, this study proposes an equity-based analysis of PT users’ travel data to estimate the real percentage of each cardholder group in sufficient detail.

Around 12 million boardings’ worth of data collected from PT-SCFC systems by KMM was used to analyze the travel behavior of users over the course of 1 month. It was found that travel and mobility pattern analysis on a large time period PT-SCFC data i.e., 1 month instead of 1-day result in more accurate determination of the real share of PT users.

To have a socially sustainable PT system, a particular and separate evaluation of the needs of users with lower frequency has to be done. PT planning according to the needs of these group may cause an increase in demand for PT. On the other hand, comparative to non-regular users, users with a higher frequency are considered, regardless of travel purposes, it seems that the current transportation service is well planned. The reason for this is that this group is already overrepresented in daily travel analyses. Frequency-based analyses not only give us the chance to evaluate transport, particularly PT, but can also assist with evaluating the impact of transport investment in terms of changing user behaviors and shifting to more sustainable transport modes. This study shows the vast extent of inequity in the number of trips used by exiting models (conventional transport planning methods). In fact, this deficiency will continue in terms of equity as long as 1-day user analysis/modeling continues to be used. This deficiency can only be eliminated if a long-time data analysis is considered in transport modeling and planning. To clarify, the limitations of existing public transport modeling and planning discussed in this study can not only be resolved by using long-time data. It is difficult to determine the real number of users without an understanding of the frequency of transport use. To address this deficit at the strategy and planning level, user categorizations ought to be constructed based on usage frequency. It can be concluded that this information should either be contained within the dataset itself or be obtained from other data (e.g., survey data). Questions over frequency of use should also consider households and other surveys conducted in SUMP [56], while Transportation Master Plan studies and user groups should be created accordingly. This solution allows for a better determination of the actual number of users. In addition, it can give important contributions at planning stage of SUMP, mainly during scenario development, measure appraisal and selection, and monitoring.

The significance of this study is not specific to Kocaeli (Turkey), but rather reveals a weakness in terms of public transportation planning in general. Further studies ought to devote more attention to examining other, more advanced models/analyses to derive more comparative groups and users’ behaviors.

Our research findings, limitation, and some recommendation for future studies are given below:

### 5.1. Research Highlights

- Card holders who have a 1–day boarding frequency represent 66% of the whole dataset, while they represent 22% in a single workday.
- Card holders with a 16–21 days boarding frequency represent 16% of the whole dataset, while they represent 39% in 1 workday.
- Regular users also have a higher boarding rate per day and will be much more overrepresented in single-day data.
- The elderly, those with disabilities, the elderly and disabled: in terms of the average number of boardings, elderly card holders (group 3), and PwD card holders (group 4) have higher (c. 10–15%) boarding rates on weekdays and weekends compared to other card holder groups. This shows that these users need more travel access. Another finding shows that PT routes and lines are not planned based on their travel needs (medical centers, elderly house, organizations for PwD, etc.), consequently, this increases their number of transfers between lines.
- Some trip routes are used by more people, even though the number of users is rarer on a daily basis, such as PT routes to medical centers (medical trips).
- Since the smart card data are boarding (transaction) based, more boardings may not really mean more trips. In other words, more boardings are likely to result from more transfer due to PT network limitations.
- Monthly users and boarding frequencies, instead of daily data, can be examined in public transportation planning, investments, improvements, and evaluated as a performance criterion.
- Travel behavior and mobility pattern of PT users varies on weekends compared to workdays due to reduced PT vehicles’ frequency (headway) and reduced number of active PT lines.

### 5.2. Limitations and Directions for Further Studies

- We proposed a novel approach using 1-month data, which can be paved the way for further studies using long-period data like yearly dataset.
- Some users use different cards or a card belong to somebody else (e.g., family members, relatives, or friends) as there is no card control (verification) or enforcement system in KMM’s PT network. Moreover, some PT users do not have a smart card or enough charge while boarding, consequently, they have to use the driver’s card or other passengers’ cards. Thus, one of the limitations of our study is the possibility of the mis-grouping of those users having low boarding frequency. This issue needs further development in the proposed method.
- It can be seen that the elderly and PwD have significantly higher boarding rates on weekdays and weekends. Using the PT boarding data for OD estimation, one may study whether this boarding rate is due to their high travel tendency or the transfer between bus lines. If it was due to their high transfer rate between the bus lines, it means transport infrastructure is not designed/planned based on vulnerable users’ group.
- In this study, 1-month PT-SCFC data has been used to analyze the travel behavior of users. The study can be extended using more than 1-month data or 1-year data to include the effects of holidays, summer seasons, etc. on the travel behavior of PT users.
- The study did not consider gender variables in clustering user groups. The gender gap and gender-responsive public transportation have been featured in many studies [57,58] and this could represent an additional point of exploration for future research.
- A simple and effective statistical analysis was conducted to come to the research hypothesis; however, advanced data analysis techniques could be employed to improve the proposed methodology. More advanced analyses may reveal more details about the nature of the phenomenon.

## Figures and Tables

**Table 1:** Modal share and trip purposed distribution [51].

Transportation Modes	Home-Based Work Trips		Home-Based School Trips		Home-Based Other Trips		Non-Home-Based Trips		Total Trips	
	No. of Trips	%	No. of Trips	%	No. of Trips	%	No. of Trips	%	No. of Trips	%
Walking	135,085	21%	539,252	70%	223,257	27%	76,573	39%	974,168	40%
Private car	172,874	21%	29,605	70%	296,496	27%	66,062	39%	565,037	23%
Shuttle service	201,878	31%	108,173	14%	13,135	2%	7397	4%	330,583	14%
Public transport	149,105	23%	89,976	12%	285,815	35%	48,230	24%	573,125	23%
Total	658,943	100%	767,005	100%	818,703	100%	198,262	100%	2,442,913	100%

**Table 2:** Original and revised card types information based on users travel behavior.

Org. Card Type	Rev. Card Type	Unique Card	# of Boarding	Org. Card Type	Rev. Card Type	Unique Card	# of Boarding
1	1	382,442	2,271,551	73	6	564	6750
10	1	16,030	79,413	78	6	521	4307
4	2	216,333	2,641,065	76	6	306	3475
65	3	57,788	425,234	79	6	225	1687
16	3	605	3804	67	6	168	2798
13	5	73,333	73,896	70	6	128	1750
5	6	22,883	148,718	72	6	87	1124
6	6	9950	104,110	77	6	46	264
69	6	3664	35,078	66	6	2	13
68	6	2203	25,918	74	4	16,254	170,436
87	6	1855	14,785	75	4	3447	20,185

**Table 3:** The distribution of card holders in weekdays and weekend.

Clustered User Groups	Boarding				Unique Card ID				Boarding Rate			
	Workday	STD	Weekend	STD	Workday	STD	Weekend	STD	Workday	STD	Weekend	STD
1: Normal	36.6%	0.6%	42.3%	0.8%	38.0%	0.5%	42.2%	0.4%	1.96	0.01	2.01	0.04
2: Student	45.9%	0.8%	40.3%	1.0%	44.9%	0.7%	39.7%	0.8%	2.08	0.02	2.04	0.03
3: Elderly	7.7%	0.3%	7.4%	0.4%	7.0%	0.3%	7.3%	0.3%	2.21	0.03	2.04	0.05
4: PwD	3.5%	0.1%	3.8%	0.2%	3.1%	0.1%	3.5%	0.2%	2.31	0.02	2.19	0.02
5: Limited Use	0.5%	0.1%	1.0%	0.2%	1.0%	0.1%	1.9%	0.3%	1.04	0.01	1.03	0.01
6: Others	5.8%	0.1%	5.1%	0.3%	5.9%	0.1%	5.3%	0.3%	1.97	0.01	1.93	0.02

**Table 4:** Workday analysis: number of boarding and users.

Trip Freq. Group	Boarding		Person	
	Workday	SDT	Workday	SDT
1-day	3.42%	0.67%	4.27%	0.74%
2-days	3.68%	0.37%	4.02%	0.38%
3-days	3.87%	0.29%	4.18%	0.30%
4-days	3.84%	0.23%	4.11%	0.23%
5-days	3.74%	0.21%	3.96%	0.19%
6-days	3.49%	0.13%	3.68%	0.13%
7-days	3.32%	0.09%	3.49%	0.08%
8-days	3.24%	0.09%	3.36%	0.09%
9-days	3.13%	0.07%	3.26%	0.07%
10-days	3.10%	0.08%	3.18%	0.09%
11-days	2.98%	0.12%	3.08%	0.12%
12-days	2.98%	0.14%	3.06%	0.15%
13-days	3.14%	0.16%	3.18%	0.17%
14-days	3.26%	0.20%	3.29%	0.19%
15-days	3.69%	0.23%	3.67%	0.22%
16-days	4.19%	0.25%	4.12%	0.23%
17-days	4.90%	0.24%	4.78%	0.23%
18-days	5.95%	0.22%	5.74%	0.22%
19-days	7.36%	0.20%	7.07%	0.20%
20-days	10.18%	0.20%	9.54%	0.19%
21-days	16.56%	0.21%	14.95%	0.18%

**Table 5:** Distribution of users and boarding frequency considering card types: daily vs. real (in a month).

Card Type	Bus Line No. 23			Bus Line No. 118		
	Person Day	TRIP DAY	Real # of Users	Person Day	Trip Day	Real # of Users
1 Normal	23.3%	24.4%	34.0%	50.1%	52.5%	55.7%
2 Students	70.7%	70.1%	56.8%	32.5%	31.1%	25.4%
3 Elderly	2.3%	2.1%	4.0%	9.1%	8.6%	10.5%
4 PwD	1.2%	1.1%	1.6%	3.4%	3.3%	3.1%
5 Limited Use	0.1%	0.1%	0.4%	0.1%	0.1%	0.3%
6 Others	2.3%	2.1%	3.4%	4.8%	4.4%	5.1%

## Data Availability

Restrictions apply to the availability of these data. Data were obtained from the Kocaeli Metropolitan Municipality and are available from the corresponding author (ghasemlou@itu.edu.tr) with the permission of the Kocaeli Metropolitan Municipality.

## Appendix A

**Table A1:** Distribution of boarding frequency of public transport (PT) users over 1-month data.

Trip Freq. Group	# of Days Which Users Have a Min. 1 Boarding	Share of Card Holder	Share of Boarding	Number of Card Holders	Share of Card holders in Entir Dataset	Avg. Boarding Per Card Holder
1-day	212,901	3.5%	2.7%	212,901	26.3%	1.57
2-days	192,544	3.2%	2.9%	96,272	11.9%	1.87
3-days	210,165	3.5%	3.2%	70,055	8.7%	1.88
4-days	214,556	3.6%	3.3%	53,639	6.6%	1.90
5-days	211,370	3.5%	3.3%	42,274	5.2%	1.91
6-days	205,074	3.4%	3.2%	34,179	4.2%	1.92
7-days	197,015	3.3%	3.1%	28,145	3.5%	1.92
8-days	188,800	3.1%	3.0%	23,600	2.9%	1.94
9-days	176,418	2.9%	2.8%	19,602	2.4%	1.95
10-days	172,460	2.9%	2.7%	17,246	2.1%	1.95
11-days	164,780	2.7%	2.6%	14,980	1.9%	1.96
12-days	162,276	2.7%	2.6%	13,523	1.7%	1.98
13-days	158,288	2.6%	2.6%	12,176	1.5%	1.98
14-days	158,466	2.6%	2.6%	11,319	1.4%	1.98
15-days	159,615	2.6%	2.6%	10,641	1.3%	2.00
16-days	168,112	2.8%	2.7%	10,507	1.3%	2.00
17-days	177,548	2.9%	2.9%	10,444	1.3%	2.01
18-days	196,038	3.2%	3.2%	10,891	1.3%	2.01
19-days	216,201	3.6%	3.6%	11,379	1.4%	2.02
20-days	251,860	4.2%	4.2%	12,593	1.6%	2.04
21-days	317,583	5.3%	5.3%	15,123	1.9%	2.05
22-days	304,788	5.0%	5.2%	13,854	1.7%	2.09
23-days	295,688	4.9%	5.1%	12,856	1.6%	2.12
24-days	273,936	4.5%	4.8%	11,414	1.4%	2.16
25-days	268,800	4.5%	4.8%	10,752	1.3%	2.18
26-days	242,554	4.0%	4.4%	9329	1.2%	2.24
27-days	189,945	3.1%	3.5%	7035	0.9%	2.26
28-days	149,324	2.5%	2.8%	5333	0.7%	2.33
29-days	114,057	1.9%	2.2%	3933	0.5%	2.39
30-days	85,200	1.4%	1.8%	2840	0.4%	2.57

**Table A2:** Distribution of users and boarding over workdays considering 1-month data vs. one-data according to their trip frequency.

	Monthly Dataset Considered				1-Day Data Considered	
Trip-Frequency Group	# of Boarding	# of Persons	Share of Boarding Per Workday	Real Share of Persons in Entire Dataset	Average Daily Boarding	Average Daily Persons
1-day	324,499	199,101	3.4%	27.7%	3.4%	4.3%
2-days	349,716	93,754	3.7%	13.1%	3.7%	4.0%
3-days	367,098	65,030	3.9%	9.1%	3.9%	4.2%
4-days	364,439	47,898	3.8%	6.7%	3.8%	4.1%
5-days	355,237	36,995	3.7%	5.2%	3.7%	4.0%
6-days	331,553	28,614	3.5%	4.0%	3.5%	3.7%
7-days	315,060	23,289	3.3%	3.2%	3.3%	3.5%
8-days	307,429	19,625	3.2%	2.7%	3.2%	3.4%
9-days	297,678	16,887	3.1%	2.4%	3.1%	3.3%
10-days	294,019	14,819	3.1%	2.1%	3.1%	3.2%
11-days	283,139	13,075	3.0%	1.8%	3.0%	3.1%
12-days	283,032	11,881	3.0%	1.7%	3.0%	3.1%
13-days	298,710	11,421	3.1%	1.6%	3.1%	3.2%
14-days	309,643	10,970	3.3%	1.5%	3.3%	3.3%
15-days	350,036	11,405	3.7%	1.6%	3.7%	3.7%
16-days	398,111	12,002	4.2%	1.7%	4.2%	4.1%
17-days	464,997	13,113	4.9%	1.8%	4.9%	4.8%
18-days	564,845	14,880	5.9%	2.1%	5.9%	5.7%
19-days	699,227	17,351	7.4%	2.4%	7.4%	7.1%
20-days	966,314	22,257	10.2%	3.1%	10.2%	9.5%
21-days	1,572,397	33,214	16.6%	4.6%	16.6%	15.0%

As seen in Table A3 and Table A4, card holders in type 1 with a 2-day trip frequency have a share of 6.79% of total boarding in workdays over the 1-month dataset. For this frequency group, which has boarding only 2 days out of 21 workdays, we do not know in which days of November they will take PT. To do so, we first calculated the percentage of the possibility of their boarding on 21 workdays of November 2018 (see table below). Then, we calculated the mean, STD, and CV for this group. The mean came to 6.79% (STD = 0.49%) with a CV value of 7.27% (which is <20%), which shows this mean (6.79%) can be properly represented for the sample group of users with 2-day boarding frequency.

**Table A3:** Workday trip-frequency distribution among the categorized user groups.

Trip Freq. Group	1 Normal		2 Student		3 Elderly		4 PwD		5 Limited-Use		6 Others	
	Board.	Person	Board.	Person	Board.	Person	Board.	Person	Board.	Person	Board.	Person
1-day	5.44%	5.77%	1.08%	1.28%	2.73%	3.37%	1.65%	2.12%	98.38%	98.70%	2.42%	2.89%
2-days	6.79%	6.98%	1.32%	1.57%	4.01%	4.78%	2.48%	3.08%	1.30%	1.08%	3.26%	3.72%
3-days	6.87%	7.06%	1.44%	1.67%	4.79%	5.54%	3.00%	3.57%	0.22%	0.17%	3.68%	4.13%
4-days	6.56%	6.69%	1.50%	1.71%	5.34%	5.98%	3.26%	3.82%	0.02%	0.02%	3.94%	4.33%
5-days	6.12%	6.21%	1.57%	1.77%	5.50%	6.08%	3.44%	3.90%	0.00%	0.00%	4.11%	4.45%
6-days	5.44%	5.51%	1.62%	1.81%	5.32%	5.77%	3.44%	3.82%	0.00%	0.00%	3.94%	4.23%
7-days	4.84%	4.89%	1.67%	1.88%	5.59%	6.00%	3.76%	4.14%	0.00%	0.00%	3.77%	4.04%
8-days	4.36%	4.36%	1.93%	2.14%	5.20%	5.42%	3.64%	3.95%	0.00%	0.00%	3.93%	4.14%
9-days	3.98%	4.02%	2.08%	2.27%	5.01%	5.13%	3.62%	3.86%	0.00%	0.00%	3.65%	3.85%
10-days	3.71%	3.74%	2.22%	2.37%	4.91%	4.91%	3.72%	3.88%	0.00%	0.00%	3.64%	3.81%
11-days	3.43%	3.50%	2.28%	2.45%	4.61%	4.63%	3.67%	3.83%	0.00%	0.00%	3.41%	3.55%
12-days	3.16%	3.23%	2.53%	2.67%	4.33%	4.30%	3.50%	3.56%	0.00%	0.00%	3.54%	3.68%
13-days	3.13%	3.17%	2.90%	3.01%	4.23%	4.12%	3.93%	3.94%	0.00%	0.00%	3.52%	3.62%
14-days	3.07%	3.13%	3.32%	3.40%	3.67%	3.47%	3.81%	3.91%	0.00%	0.00%	3.40%	3.53%
15-days	3.23%	3.27%	4.02%	4.04%	3.86%	3.67%	4.17%	4.05%	0.00%	0.00%	3.70%	3.79%
16-days	3.42%	3.43%	4.87%	4.84%	3.92%	3.60%	4.22%	4.10%	0.08%	0.04%	4.41%	4.39%
17-days	3.60%	3.62%	6.11%	5.99%	3.80%	3.50%	4.70%	4.77%	0.00%	0.00%	5.50%	5.40%
18-days	4.05%	3.93%	7.92%	7.79%	4.03%	3.66%	5.34%	5.10%	0.00%	0.00%	5.73%	5.63%
19-days	4.63%	4.48%	10.40%	10.19%	4.12%	3.63%	6.24%	5.97%	0.00%	0.00%	6.17%	5.87%
20-days	5.62%	5.30%	14.95%	14.39%	5.68%	4.89%	9.05%	8.38%	0.00%	0.00%	8.69%	7.82%
21-days	8.55%	7.69%	24.25%	22.76%	9.35%	7.52%	19.36%	16.27%	0.00%	0.00%	15.60%	13.13%

**Table A4:** Distribution of share of boarding over a month.

Date of November 2018	1	2	3	4	5	6	7	8	9	10	11	12	13	14	15
Share of boarding (trip)	7%	5%	5%	5%	6%	9%	9%	5%	4%	4%	5%	5%	8%	9%	5%
Date of November 2018	16	17	18	19	20	21	22	23	24	25	26	27	28	29	30
Share of boarding (trip)	4%	5%	5%	6%	8%	9%	5%	5%	5%	5%	7%	9%	10%	10%	9%
